# In Memoriam: Myron Gilbert Schultz (1935–2016)

**DOI:** 10.3201/eid2508.190356

**Published:** 2019-08

**Authors:** David M. Morens, Rohit A. Chitale

**Affiliations:** National Institutes of Health, Bethesda, Maryland, USA (D.M. Morens);; Associate Editor, Emerging Infectious Diseases, Atlanta, Georgia, USA (D.M. Morens);; PATH, Seattle, Washington, USA (R.A. Chitale)

**Keywords:** Global health, zoonoses, zoonotic diseases, epidemiology, HIV, AIDS, public health, tropical medicine, medical history, parasitic diseases, surveillance, event-based surveillance

In 1954, a freshman veterinary student became “engaged in an inner struggle” (*1*). A wise mentor took him to hear a minister speak about the missionary physician Albert Schweitzer (1875–1965), who had devoted his entire life to serving others. “I was astonished that such a man could exist,” the student remembered (*1*). Each morning the student repeated an 18th century prayer reflecting the teachings of physician-philosopher-rabbi Moses ben Maimon, or Maimonides (1135–1204): “Grant that I may be filled with love for my art and for my fellow man. May the thirst for gain and the desire for fame be far from my heart” (*2*). With renewed purpose, the young scholar finished his Doctor of Veterinary Medicine degree (1958), then his Medical Doctor degree (1962). Thus began the remarkable, humanistic career of Myron Gilbert (“Mike”) Schultz (Figure). Sixty years later, that career still flourished in humanistic faith.

**Figure:**
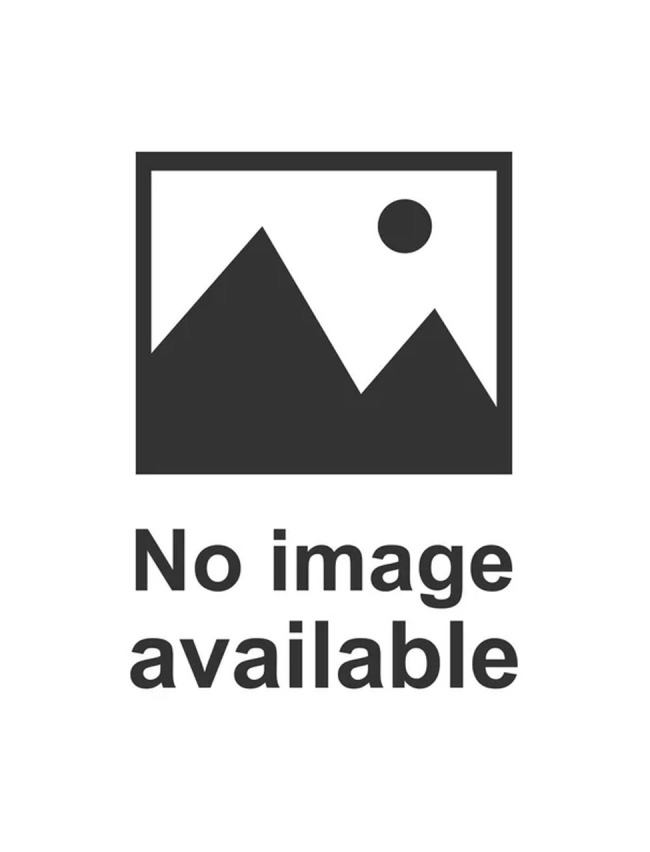
Myron Gilbert “Mike” Schultz (1935–2016). His career in global public health and zoonotic disease control spanned 53 years, almost entirely at the Centers for Disease Control and Prevention (CDC).

Myron Gilbert Schultz, DVM, MD, DCMT, FACP, was born to middle-class parents in the Bronx, New York City, New York, USA, on January 6, 1935. After graduation from the Bronx High School of Science, he spent 2 years (1952–1954) at the New York State College of Agriculture, 4 more at the New York State College of Veterinary Medicine, Cornell (1954–1958), and another 4 at Albany Medical College (1958–1962), during which time he supported himself by practicing veterinary medicine at the Saratoga Raceway and, as he would later relate, repeatedly abandoning the horses to rush back and deliver babies.

With DVM and MD degrees in hand, Mike interned at the US Public Health Service Hospital (Boston, MA, USA). This internship led to his recruitment by Alexander D. Langmuir (1910–1993) and a transformative 2-year stint in Langmuir’s Atlanta-based Epidemic Intelligence Service (EIS) training program at the (then-named) Center for Disease Control (CDC). Mike’s EIS experiences included a 1964 deployment to Vietnam to investigate infectious disease threats in the war and an important friendship with James Harlan Steele, DVM (1913–2013), the renowned veterinary epidemiologist/epizootiologist whose leadership helped to formulate their shared concept of “One Health”—the idea that humans, animals, and the environment are all part of an intertwined ecosystem with respect to disease occurrence and microbial evolution—and to shape the conceptualization of emerging infectious diseases (*3*). At the end of those 2 years in EIS, Mike was held in such regard that he was sent off on 2 successive career development assignments: an infectious diseases fellowship at New York City’s Bellevue Hospital (1965–1966) and another year at the London School of Hygiene and Tropical Medicine (1966–1967).

At just 32 years of age, Mike returned to CDC to become chief of the newly created Parasitic Diseases Branch (1967–1973), and when the branch was elevated to a division, he was named its director (1973–1982). These were years of extraordinary productivity. Almost immediately after becoming branch chief, Mike began studying the national epidemiology of *Pneumocystis carinii* (now named *P. jirovecii*) pneumonia (*4*,*5*), which, 14 years later, would provide the first evidence of the AIDS epidemic (*6*). He established human giardiasis as an important endemic and travel-associated disease (*7*); contributed significantly to the new field of travel medicine (*8*,*9*), initiating the “Yellow Book” of health information for travelers; co-developed the Field Epidemiology Training Program to teach epidemiology around the world; created CDC’s Parasitic Diseases Drug Service, bringing to patients worldwide important drugs, such as pentamidine to treat African trypanosomiasis; studied parasitic disease outbreaks in Micronesia, in Native Americans, and in US mental institutions; chaired the first international symposium on dracunculiasis (Guinea worm disease), now close to eradication; and dealt with a host of other parasitic diseases directly and through leadership of a talented cadre of CDC scientists and EIS officers (*10*–*13*).

During this same time, Mike also displayed great talent as a medical historian, evidenced by a rigorously researched special article in the New England Journal of Medicine about Daniel Carrión’s 1885 elucidation of bartonellosis (*14*); medical detective work on Robert Louis Stevenson’s creation of Dr. Jekyll and Mr. Hyde (*15*); and, most memorably, lectures and publications about US Public Health Service epidemiologist József (known as Joseph) Goldberger (1874–1929) (*16*), who discovered the mode of acquisition of pellagra. (D.M.M. remembers Mike’s utter delight when, in 1976, he learned that an incoming member of that year’s EIS class, Mark Goldberger, MD, was none other than the great-great-nephew of Joseph Goldberger). Mike wrote with reverence about 2 additional giants of US epidemiology who pioneered the concepts of emerging infectious diseases, Theobald Smith (1859–1934) and Calvin Schwabe (1927–2006); his later series of Photo Quiz essays published in Emerging Infectious Diseases—each essay based on identifying a photograph of a scientist who had made important contributions—taught a new generation about the field’s history (Appendix Table). Mike displayed yet another talent: writing elegant memoriams upon the deaths of admired scientists, such as malariologist Meir Yoelli (1912–1975) (*17*).

Within Mike’s first decade at CDC, the pattern of his work and ethical approach had been set: hard work, a high level of accomplishment, a humanistic outlook, concern for less fortunate persons, and a desire to improve lives through medicine and public health. He gave to others without seeking reward or recognition. He respected heroism and sacrifice—not only the sacrifices of men like Schweitzer but also men like Carrión, who died after inoculating himself to discover the cause of bartonellosis (*14*); like Goldberger, the Eastern European immigrant who overcame oppressive anti-Semitism (*16*); and even like Stevenson, who abandoned his world of comfort to die of tuberculosis on a remote Pacific island among beloved native Samoans (*18*). Mike cared greatly about migrants and refugees; his essay on forgotten diseases, an early formulation of what are now called neglected tropical diseases, was as much about forgotten people as about forgotten diseases (*19*).

Some said Mike was shy, but those who knew him well would disagree. D.M.M. recalls a co-authored biographical sketch about microbiologist-epidemiologist Charles Nicolle (1866–1936) (*20*). In an exchange of manuscript drafts, D.M.M.’s intended upgrade of a paragraph on Nicolle’s probable isolation of the 1918 influenza pandemic virus was firmly rebuffed: Nicolle’s elegant work elucidating the cause of typhus made him great, Mike insisted; Nicolle’s other accomplishments were subordinate and needed to stay that way—and they did.

Mike was still restlessly productive and engaged when in 2008 he joined CDC’s Global Disease Detection program, in what would be his last professional position. Mike was attracted to new challenges, which the young program provided. Work in the event-based surveillance unit, located inside CDC’s Emergency Operations Center, was fast-paced and often frenetic, and it should have been too much for a man of advancing years. But Mike embraced the work and even thrived in its stressful team environment. He still saw the world with an almost childlike wonder; he was fascinated by the new public health approaches and technologies. R.A.C. recalls Mike’s delight that, through CDC’s disease detection systems, 1 dead cow could be identified in the middle of Saudi Arabia—a harbinger of a potential zoonosis. Voicing the apocryphal “Chinese curse” (actually a mid-20^th^-century saying of disputed English language origin), Mike would comment that “We live in interesting times!,” a wry observation he repeated over many years and through many different challenges.

And so it seemed like the end of a noble era when Mike Schultz died, at age 81, on February 19, 2016. Those who mourned his passing spanned 3 generations, from each continent, across many disciplines, including a large loving family and a host of cherished friends and colleagues. The remembrances that poured forth, written independently by many different people, were strikingly alike in describing Mike as “selfless,” “dedicated,” and possessing “passion, wisdom, and patience.” The New York Times described Mike’s identification of the AIDS epidemic (*21*), but Mike unselfishly credited colleague Sandy Ford (1950–2015) as having noticed, in 1981, increased pentamidine requests for desperately ill men (*6*), representing a first important clue to uncovering AIDS. Nor did Mike mention his many prestigious awards, including 2 Public Health Service Meritorious Service Awards, CDC’s William C. Watson, Jr., Medal of Excellence, or the Bailey K. Ashford Medal from the American Society of Tropical Medicine and Hygiene, for which he credited his mentors and his wife, Selma, for having inspired him to work to “end the suffering… that ruins the lives of hundreds of millions of people…” (*22*).

The London School of Hygiene and Tropical Medicine remembers Mike’s Frederick Murgatroyd award as best student and for the renowned mentors who became lifelong friends, including Ben Kean (1912–1993) and Leonard Bruce-Chwatt (1907–1989), head of the (Sir Ronald) Ross Institute (*23*). The American Veterinary Medical Association noted Mike’s leadership in parasitology, his co-founding of CDC’s Field Epidemiology Training Program, and his leadership in developing the clinical specialty of travel medicine (*24*). Mike’s life work bound together the fabric of global public health over >5 decades.

However, the legacy of Mike Schultz is not only one of awards, publications, and accomplishments but also of thousands of acts of generosity, given freely to all, in the capacity of mentor, friend, humanitarian, philosopher, and lover of knowledge. Whatever was going on at CDC, indeed in global public health, Mike was likely to be there, comfortably in the shadows, helping, teaching, encouraging and praising others, leading quietly and by example, showing how the rigor of science and the humanism of the healing arts could be brought to those who most needed help.

What more can be said about Mike? He was a devoted family man and an imaginative artist: his award-winning sculpture, Galatea, once on display at the Atlanta Memorial Art Center, not only moved but also emitted the recorded notes of the Bachianas Brasileiras No. 5 of composer Heitor Villa-Lobos (1887–1959). He was quietly proud of his scientific and artistic work but embarrassed by adulation, uncomfortable with attention to his virtues. We share 2 unrelated observations that describe Mike well. After his death, a colleague wrote in the West African language Yoruba that Mike was “an omolubi, a person of honor who believes in hard work, respects the rights of others, and gives to the community in action and deeds, a person of integrity… a man of character.” In 1997, Mike himself wrote the following words upon the death of his close friend, Meir Yoelli: “In his company, ordinary things were transfigured; they became romantic and acquired great import… I watched him bring a moment of joy to a shoeshine man…. In his presence… the world was no longer prosaic... He was, without exception, the finest man I have ever known” (*17*).

In aspiring to live up to the humanitarian ideals of men like Yoelli, Goldberger, and Schweitzer, Mike Schultz lived a life of richness and meaning; he touched countless others with his gentle spirit, his faith, and his belief in their inherent worthiness, inspiring them to find their own best selves. He now sits quietly in the company of his great heroes, and we remember him as one of them.

Appendix Photo quiz articles authored by Myron G. Schultz, DVM, MD, that appeared in Emerging Infectious Diseases.
