# Supplementary material for: In Memoriam: Myron Gilbert Schultz (1935–2016)
Source: Emerg Infect Dis. 2019 Aug;25(8):1617–9. doi: 10.3201/eid2508.190356 (PMC6649340; doi:10.3201/eid2508.190356)
Supplement: Appendix — Photo quiz articles authored by Myron G. Schultz, DVM, MD, that appeared in Emerging Infectious Diseases. [file 19-0356-Techpp-s1.pdf]

Article DOI: https://doi.org/10.3201/eid2508.190356

In Memoriam: Myron Gilbert Schultz (1935–2016)

Appendix

Appendix Table. Photo quiz articles authored by Myron G. Schultz, DVM, MD. that appeared in Emerging Infectious Diseases

Scientist profiled	Date published	Link to quiz	Co-author
Rudolf Virchow	2008 Sep	https://wwwnc.cdc.gov/eid/article/14/9/pdfs/08-6672.pdf	None
Theobald Smith	2008 Dec	https://wwwnc.cdc.gov/eid/article/14/12/pdfs/08-1188.pdf	None
Charles-Jules-Henri Nicolle	2009 Sep	https://wwwnc.cdc.gov/eid/article/15/9/pdfs/09-0891.pdf	David M. Morens
Henry Rose Carter	2009 Oct	https://wwwnc.cdc.gov/eid/article/15/10/pdfs/09-0129.pdf	None
Daniel Alcides Carrión	2010 Jun	https://wwwnc.cdc.gov/eid/article/16/6/pdfs/09-1937.pdf	None
Robert Koch	2011 Mar	https://wwwnc.cdc.gov/eid/article/17/3/pdfs/10-1881.pdf	None
Calvin W. Schwabe	2011 Dec	https://wwwnc.cdc.gov/eid/article/17/12/pdfs/11-0484.pdf	Peter Schantz
Alexander Duncan Langmuir	2015 Sep	https://wwwnc.cdc.gov/eid/article/21/9/pdfs/14-1445.pdf	William Schaffner
